# Human Odorant Reception in the Common Bed Bug, *Cimex lectularius*

**DOI:** 10.1038/srep15558

**Published:** 2015-11-02

**Authors:** Feng Liu, Nannan Liu

**Affiliations:** 1 Department of Entomology and Plant Pathology, Auburn University, Auburn, AL 36849, USA

## Abstract

The common bed bug *Cimex lectularius* is a temporary ectoparasite on humans and currently resurgent in many developed countries. The ability of bed bugs to detect human odorants in the environment is critical for their host-seeking behavior. This study deciphered the chemical basis of host detection by investigating the neuronal response of olfactory sensilla to 104 human odorants using single sensillum recording and characterized the electro-physiological responses of bed bug odorant receptors to human odorants with the *Xenopus* expression system. The results showed that the D type of olfactory sensilla play a predominant role in detecting the human odorants tested. Different human odorants elicited different neuronal responses with different firing frequencies and temporal dynamics. Particularly, aldehydes and alcohols are the most effective stimuli in triggering strong response while none of the carboxylic acids showed a strong stimulation. Functional characterization of two bed bug odorant receptors and co-receptors in response to human odorants revealed their specific responses to the aldehyde human odorants. Taken together, the findings of this study not only provide exciting new insights into the human odorant detection of bed bugs, but also offer valuable information for developing new reagents (attractants or repellents) for the bed bug control.

The common bed bug, *Cimex lectularius,* is a temporary hematophagous ectoparasite on human being and animals^1^,^2^, with all its developmental stages and both sexual forms relying on blood sources for nutrition and reproduction. Although virus transmission has been rarely reported for *C. lectularius*, the bite nuisance and potential for secondary infections create both physical and psychological disturbance in human hosts^3^. The introduction of effective chemical insecticides removed the common bed bug as a subject of public concern for many years as populations were controlled and almost eradicated in some industrialized countries^3^. However, in the early 21^st^ century the common bed bug was reported to be resurgent, causing serious problems for public health^4^,^5^,^6^,^7^. The resurgence of the common bed bug led to a search for new sustainable methods to monitor and control this human ectoparasite. Regarding to increased insecticide resistance of bed bugs, traps baited with attractive cues represent a promising complementary method in the bed bug control. As in other blood-feeding insects such as mosquitoes, human odorants possess great potential as attractant to the bed bugs. Indeed, previous studies of behavioral response to human volatiles have revealed that human sweat alone has a significant attraction for all stages and both sexes of bed bugs^8^ and other studies have indicated that odors from animal skin emanations are also attractive to bed bugs^9^,^10^.

The olfaction system of bed bugs plays an important role in their host-seeking process. Olfactory receptor neurons housed in olfactory sensilla on bed bug antennae are responsible for detecting human odors^11^. The odorant receptors on the neuron membrane bind to human odors which will result in the depolarization of the neuron membrane and the production of action potentials^12^,^13^,^14^,^15^. Three types of olfactory sensilla (C, D, E sensillum) on the bed bug antennae have been morphologically identified by Levinson *et al.* (1974)^16^. More recently, functional studies have further categorized the D type of sensillum into three types, Dα, Dβ, and Dγ, based on their distinctive response profiles to the chemicals in single sensillum recording (SSR)^17^,^18^.

Despite the promising application in the bed bug control, only a few human odorants have been tested on bed bugs using single sensillum recording or in the behavior assays^11^. In an effort to characterize the interaction between the bed bug olfactory sensillum and human odorants and decipher the molecular basis of odorant detection by the bed bug olfactory system, we conducted a systematic characterization of the neural responses of bed bug olfactory sensilla to 104 commercially available human odorants using the single sensillum recording and, for the first time, reveal the functions of two bed bug odorant receptors in response to these odorants using the *Xenopus* expression system coupled with Two-Electrode Voltage Clamp.

## Results

### Response, tendency and tuning curve of olfactory sensilla to human odorants

**Figure 1:**
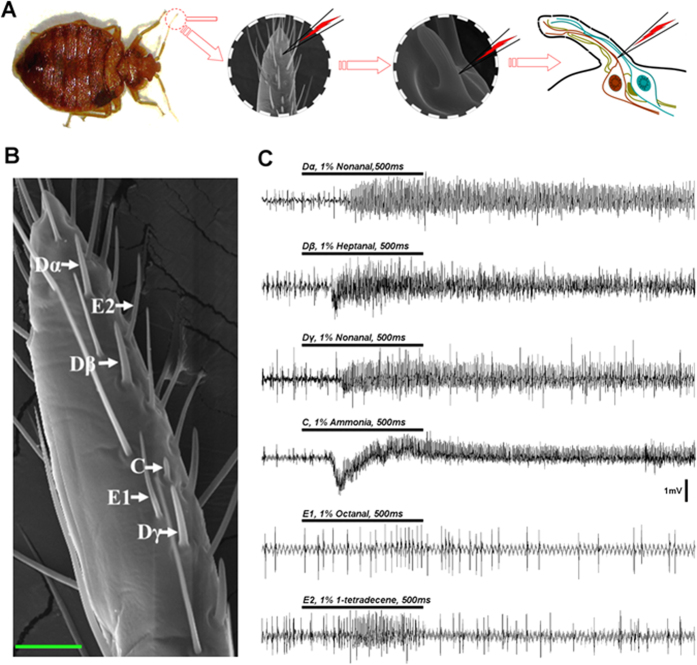
Single sensillum recording on different types of olfactory sensilla in the common bed bug, *C. lectularius*. (**A**) Schematic image of single sensillum recording in the olfactory sensilla on bed bug antennae. (**B**) SEM photo (modified from Liu *et al.*, 2014) showing the different types of olfactory sensilla on bed bug antennae. The scale bar indicates 20 μM. (**C**) The highest neural responses for each type of olfactory sensillum to different human odorants.

We tested the neural responses of each type of olfactory sensilla to 104 chemicals from 11 chemical groups utilizing single sensillum recording (Fig. 1A) and found that different sensilla (Fig. 1B) displayed markedly different characteristic neuronal responses to different human odors. In general, each type of sensilla exhibited its highest exciting response to a different odorant, with the exception of Dα and Dγ, which showed the strongest response to the same human odor, namely nonanal (Fig. 1C).

**Figure 2:**
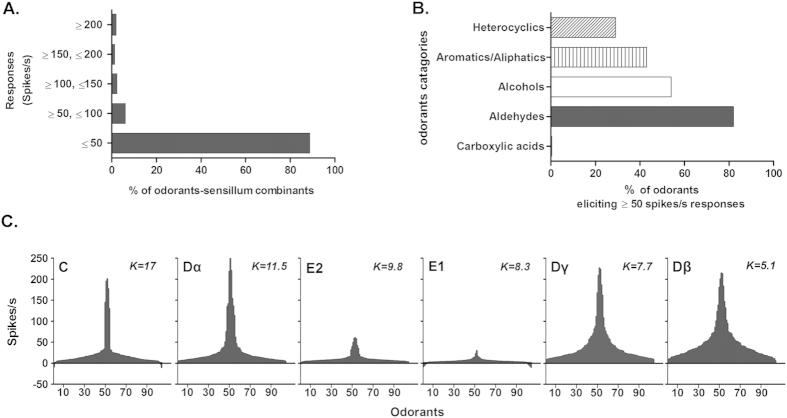
Summary of the responses of olfactory sensilla in the common bed bug, *C. lectularius*, to human odorants. (**A**) Distribution of firing frequencies for different strengths of responses to different odorant/sensillum combinations; (**B**) Response biases to different odorant categories with firing frequencies higher than 50 spikes/s. Sensilla that failed to show a response ≥15 spikes/s were considered non-responders. The excitatory response of 50 spikes/s was selected as the criterion which represents a 20% increase of the largest firing frequency recorded (248.5 spikes/s, for nonanal in Dα sensilla). (**C**) Different tuning curves of olfactory sensilla for human odorants. The 104 odorants are distributed along the x axis according to the strengths of the responses they elicited from each sensillum. The odors that elicited the strongest responses are near the center of the distribution; those that elicited the weakest responses are near the edges. The order of the odorants therefore differs for different sensilla. Negative values indicate inhibitory responses. The kurtosis value, K value, as a statistical measure of ‘peakedness’, is shown on the right side for each plot.

In total, 624 odorant-sensillum combinations, with each chemical being tested for all six types of sensilla, were recorded with at least six replicates on different individuals. Of these combinations, 88.8% (554) of the odorant-sensillum combinations yielded little if any response (<50 spikes/s); 6.1% (38) resulted in responses of ≥50, ≤100 spikes/s; 2.2% (14) produced a strong response of ≥100, ≤150 spikes/s; 1.3% (8) resulted in very strong responses of ≥150, ≤200 spikes/s; and 1.6% (10) generated extremely strong responses of ≥200 spikes/s (Fig. 2A). This result indicates that strong or even mild neuronal responses to human odorants are actually sparse.

To investigate whether the common bed bugs had special tendencies or biases towards detecting particular types of human odorants, we compared the ratios of the major chemical groups (carboxylic acid, aldehydes, alcohols, aromatic/aliphatics, and heterocylics) that elicited excitatory responses (≥50 spikes/s) in the bed bugs and found that they responded to 82% of the aldehydes, 50% of the alcohols/aliphatics, 40% of the aromatics, and 30% of the heterocyclics (Fig. 2B). Interestingly, although carboxylic acids make up the largest chemical group of the human odorants tested, 21 of the 104 chemicals, none of the bed bugs’ olfactory antennal sensilla had an excitatory response of ≥50 spikes/s to any carboxylic acid (Fig. 2B). This distinctive differentiation of neuronal responses in the common bed bugs to human odorants suggests that certain chemical groups (such as the aldehydes) may play a key role in the host-seeking process of bed bugs.

**Figure 3:**
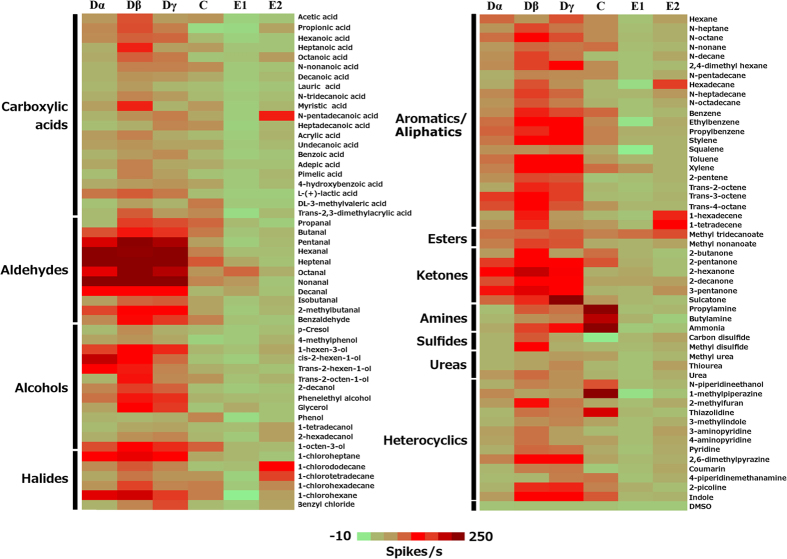
A heatmap presentation of the responses of olfactory sensilla of the common bed bug*, Cimex lectularius*, to human odorants. Distinctive response profiles (spikes/s) of Dα, Dβ, Dγ, C, E1 and E2 sensilla to different chemical groups of human odorants were tested through single sensillum recording, with at least six replicates for each odorant on different individual sensilla at a dose of 1:100 v/v. The solvent, DMSO, produced no stimulation in any of the sensilla types.

The tuning curves of the neuronal responses revealed the preference for each type of olfactory sensillum in detecting semiochemicals in the environment. Within the six different types of olfactory sensillum on the bed bug antennae, the tuning curve ranged from extremely narrow (in the C sensillum with a K value of 13) to very broad (in the Dβ sensillum with a K value of 5.1), displaying a continuous pattern (Fig. 2C). The narrowly tuned C sensillum responded to only a few chemicals, mostly amines with very high firing frequencies, while the most broadly tuned Dβ sensillum responded strongly to human odorants with very diverse chemical structures (Fig. 3). This difference among the tuning curves for different types of olfactory sensillum indicates their potential capacity in detecting the odorants from human host. Particularly, based on their broad tuning curve to human odorants in this study and also supported by the findings in previous studies^17^,^18^, we conclude that the D type olfactory sensilla, particularly Dβ and Dγ, play the predominant role for the bed bug to detect chemical stimuli in the environment, including the human odors.

### Olfactory responses of D sensilla to the human odorants in *C. lectularius*

As noted above, the D sensilla (Dα, Dβ and Dγ) play the most important role in detecting the major chemical groups (aldehydes, alcohols, heterocyclics, and aromatics/aliphatics) in human odorants, far outpacing the other types of olfactory sensilla. Specifically, Dα sensilla responded to 55%, 15% and 15% of the aldehydes, alcohols, and heterocyclics, respectively, with a firing frequency of ≧50spikes/s; Dβ sensilla responded to 82%, 38% and 30% of the aldehydes, alcohols, and aromatics/aliphatics, respectively, with a firing frequency of ≧50spikes/s; and Dγ sensilla responded to 64%, 26% and 15% of the aldehydes, aromatics/aliphatics, and heterocyclics, respectively, with a firing frequency of ≧50spikes/s.

Interestingly, the D sensilla also showed strong responses to a few chemicals in the minor chemical groups (ketones, halides, etc.) in human odorants. For example, the Dα sensilla reacted to one of the halides (1-chlorohexane) with a neuronal response of 136 ± 13.59 spikes/s (Fig. 3, Table S1). The Dβ sensilla also showed strong excitatory responses to two halides (1-chloroheptane and 1-chlorohexane) with firing rates of 146 ± 12.59 and 131 ± 3.53 spikes/s, respectively (Fig. 3, Table S1). Moreover, both the Dβ and Dγ sensilla were very sensitive to several ketones. Dβ sensilla showed strong responses to 2-pentanone, 2-hexanone, 2-decanone and 3-pentanone, with firing rates of 102 ± 4.8, 138 ± 9.12, 100 ± 5.2, 122 ± 4.8 spikes/s, respectively, while the Dγ sensilla showed strong responses to 2-hexanone and sulcatone, with firing rates of 111 ± 6.1 and 226 ± 7.36 spikes/s, respectively (Fig. 3, Table S1).

### Olfactory responses of C, E1 and E2 sensilla to the human odorants in *C. lectularius*

The grooved peg C sensilla (nine on each antenna) each house 4–5 sensory neurons, and these were found to exhibit a much lower sensitivity to most of the human odorants tested than the smooth peg D sensilla. C sensilla revealed no systematic response to several of the major chemical groups in human odorants, including carboxylic acids, aldehydes, alcohols and aromatics/aliphatics. However, the grooved peg C sensilla did exhibit systematic sensitivity to amines, including ammonia, propylamine, and butylamine, with firing frequencies of 200 ± 6.97, 195 ± 15.93, and 144 ± 12.06 spikes/s, respectively (Table S1). Two additional heterocyclics, 1-methylpiperazine and thiazolidine, were also found to be strong stimuli for the C sensilla, with firing rates of 176 ± 41 and 130 ± 36 spikes/s, respectively (Table S1).

The hair-like E sensilla are the most abundant sensilla on bed bug antennae, although they house far fewer sensory neurons (1–3 sensory neurons) and pores on the sensilla cuticle compared to the D and C sensilla. In this study, two types of E sensilla, E1 and E2, exhibited very different neuronal signals. The E1 sensilla did not respond to any of the human odorants except for weak responses to two chemicals, octanal and methyl tridecanoate, with firing frequencies of 30 ± 4.45 and 23 ± 2.93 spikes/s, respectively (Table S1). However, the E2 sensilla showed much greater activity in response to the long-chain chemicals in human odorants. Marked excitatory responses were observed in the E2 sensilla in response to several human odorants, four of which, N-pentadecanoic acid, 1-tetradecene, lauroyl chloride and 1-chlorododecane, elicited responses with firing frequencies of 49 ± 4.4, 60 ± 5.63, 58.80±6.00 57 ± 9.49 spikes/s, respectively (Table S1). The E2 sensilla also show weaker responses to another five human odorants, namely hexadecane, 1-hexadecene, methyl tridecanoate, 1-chlorotetradecane, and 1-chlorohexadecane, with neuronal responses of 35 ± 3.5, 42 ± 5.29, 31 ± 2.93, 39 ± 4.6, 20 ± 1.38 spikes/s, respectively (Table S1). Since all the chemicals that generated responses from the E2 sensilla possess more than ten carbons in their molecular backbone, it seems likely that the E2 sensilla on the common bed bug antennae are responsible for detecting the long-chain chemicals in human odorants.

### Dose dependent responses of olfactory sensilla to human odorants

**Figure 4:**
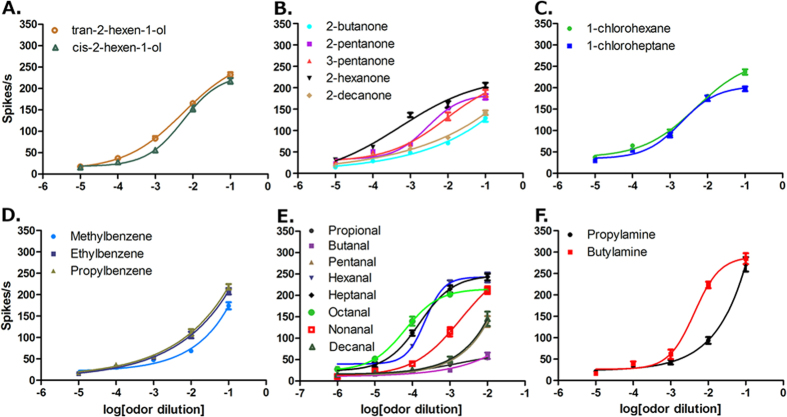
Dose-dependent responses of bed bug olfactory sensilla to human odorants. The dose-dependent response curve is presented as a mean value ± SEM, n ≥ 6. (**A**) Dose-dependent response of Dα sensilla to two stereoisomers of 2-hexen-1-ol; (**B**) Dose-dependent response of Dβ sensilla to ketones; (**C**) Dose-dependent response of Dβ sensilla to halides; (**D**) Dose-dependent response of Dγ sensilla to aromatics; (**E**) Dose-dependent responses of Dγ sensilla to aldehydes; and (**F**) Dose-dependent response of grooved peg C sensilla to two amines, propylamine and butylamine. The X axis describes the logarithm dilution series from 1:10 to 1:10^5^ v/v in (**A**–**F**) and from 1:10^2^ to 1:10^6^ v/v in (**E**).

To investigate the effect of chemical dosage on the neuronal responses of olfactory sensilla to human odorants, the responses of Dα, Dβ, Dγ and C sensilla to different doses were tested. Human odorants that had previously shown strong stimulations at a 100-fold dilution (v/v) were chosen for this dose-response study. Basically, all different types of olfactory sensilla tested showed a dose-dependent response to the human odorants. One particularly interesting result was the comparison of two alcohols, trans-2-hexen-1-ol and cis-2-hexen-1-ol, with Dα sensilla, where the results showed that as the doses increased from 1:10^5^ to 1:10 v/v, the neuronal response of Dα sensilla to both chemicals increased accordingly, rising from ≤20 spikes/s to ≥200 spikes/s (Fig. 4A). The very similar dose-dependent curves may result from their similar chemical structures.

A number of ketones (2-pentanone, 2-butanone, 2-hexanone, 2-decanone and 3-pentanone) and halides (1-chlorohexane and 1-chloroheptane) were also chosen for the dose-response test for the Dβ sensilla, which displayed the highest firing frequency to these human odorants at the original dose of 1:10 v/v. Here, the lowest dose-response curve was observed in 2-butanone and the highest in 2-hexanone (Fig. 4B). The 2-pentanone/3-pentanone and 1-chlorohexane/1-chloroheptane pairs showed quite similar dose-dependent stimulation for the Dβ sensilla at different doses (Fig. 4B,C), which make quiet sense based on their closing chemical structures.

For the Dγ sensilla, aromatics (ethylbenzene, propylbenzene, and methylbenzene (toluene)) and aldehydes (from propanal to decanal) were chosen for the dose-response tests. All these human odorants showed their strongest stimulation on the Dγ sensilla compared to other types of sensilla at the original dose of 1:10 v/v. For the three aromatic human odorants, the Dγ sensilla showed statistically significantly stronger responses to ethylbenzene and propylbenzene compared with methylbenzene (F test, P < 0.0001) (Fig. 4D). For the aldehydes, hexanal, heptanal and octanal generated the strongest stimulations with the threshold of responses at least one-log dose lower than nonanal, two-log doses lower than decanal and pentanal and three-log doses lower than propional and butanal (Fig. 4E).

The two human odorants that showed the strongest stimulation on the grooved peg C sensilla, propylamine and butylamine, were chosen to conduct the dose-response test for the C sensilla. The C sensilla displayed quite similar responses to both amines, with no statistically significant differences in the responses at doses of 10^5^, 10^4^, 10^3^ and 10-fold dilution (v/v) (*t* test, P > 0.05). However, at the 10^2^-fold dilution (v/v) doses, the firing frequency of C sensilla to butylamine (223 ± 20 spikes/s) was significantly higher than that for propylamine (94 ± 20 spikes/s) (*t* test, P < 0.001) (Fig. 4F). Taken together, these results indicated that the specific dosage of human odors is very important in triggering the olfactory neural responses of bed bugs to their hosts.

### Temporal dynamics of olfactory sensilla in response to human odorants

**Figure 5:**
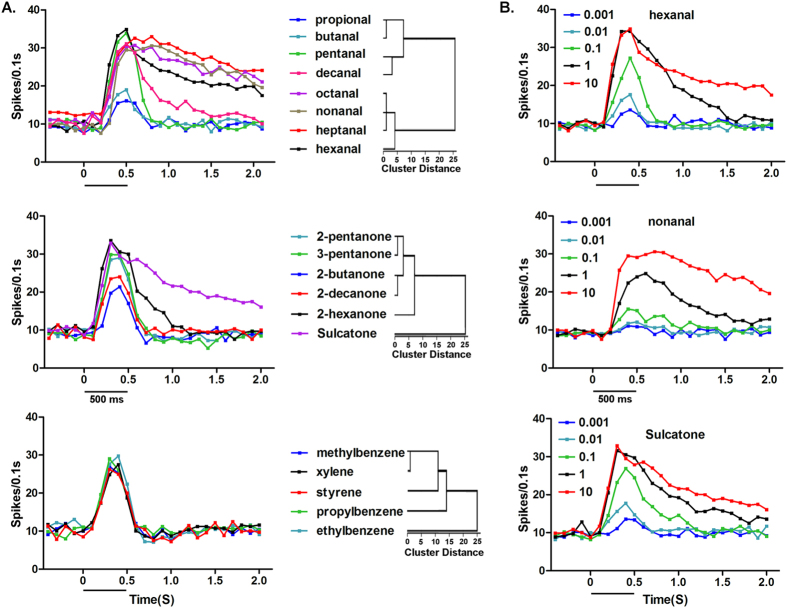
Temporal dynamics of olfactory sensilla in response to human odorants. (**A**) Temporal structures of neuronal responses of Dα sensilla in response to aldehyde, ketone and aromatic odorants at a dose of 1:100 v/v. The left side of the figure shows a trace representing the mean value of spikes (n = 8, error bars are not shown) recorded during each 100 ms sampling period. The right side of the figure shows the hierarchical cluster analysis for the odorants, with the corresponding categories based on the action potential number in each single 100 ms sampling period. (**B**) Temporal structures of dose-dependent responses of Dα sensilla in response to hexanal, nonanal and sucaltone at doses ranging from 1:10^2^ (10 µg/µL) to 1:10^6^  (0.001 µg/µL) v/v. Horizontal bars indicate the duration of the stimulation (500 ms).

Besides the firing frequency, the temporal structure of olfactory neural responses, is considered to be another important factor involved in the odor coding process^19^,^20^,^21^,^22^. To investigate the temporal structure of these neural responses in the bed bug, we examined the firing frequencies of the olfactory sensilla over a 2 s period beginning at the onset of chemical stimulation. Responses were plotted onto a continuous line graph at 100 ms intervals. The results show that the temporal characteristics of ORNs in the olfactory sensillum are indeed both stimulus and dose specific. For instance, the temporal structure of the Dγ sensillum’s response to aldehydes at a dose of 1:100 v/v varied considerably (Fig. 5A). Propional, butanal and decanal were more likely to elicit a phasic neuronal response, while pentanal, hexanal, heptanal, octanal and nonanal instead tended to generate a tonic neuronal responses, with the firing rates remaining at a high level 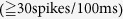 throughout the 2 s time period. Sulcatone was the only ketone that presented a tonic response; all the others (2-hexanone, 2-pentanone, and 2-decanone) displayed more phasic responses from the olfactory neurons (Fig. 5A). Aromatic chemicals generally elicited phasic neuronal responses at a dose of 100-fold dilution (v/v), with no typically tonic responses observed (Fig. 5A). The cluster analysis based on the temporal structures of these neural responses further distinguished human odors with the same categories. For example, the aldehydes (C3–C10) were evidently separated into two groups just according to their neural temporal structure differences (Fig. 5A). The same thing was applied to ketones, among which sucaltone was obviously discriminated from the aliphatic ketones, perhaps resulted from their quite different molecular structures (Fig. 5A). For the aromatics, ethylbenzene was also slightly set apart from other aromatics with a relatively short cluster distance (Fig. 5A). In conclusion, the wide variations in the temporal structures of neural responses may influence further odorant recognition for the bed bugs.

Furthermore, the temporal dynamics of neuronal responses were also significantly influenced by the odor dosages or intensity. Low doses of human odors appeared to generate more phasic neuronal responses, while high doses were more likely to elicit typically tonic responses from the olfactory neurons. For some human odors, like hexanal, nonanal and sulcatone, the firing processes were prolonged greatly as the doses increased from 10^6^-fold to 10^2^-fold dilution (v/v) and the temporal dynamics shifted from predominantly phasic to become more tonic (Fig. 5B), which was also the case for several other efficient stimuli, including heptanal, octanal, 1-chloroheptane and 1-chlorohexane (Data not shown).

### Primary presentations of odorant space among the olfactory sensilla

Our results clearly showed that different human odors elicit different patterns of response combinations from different bed bug olfactory sensilla. To investigate the ability of bed bugs to differentiate between human odors in different categories, we examined the primarily spatial relationships among odorants in an odorant space created by the responses of each olfactory sensillum to each of the odorants tested. In this six-dimensional odorant space, Euclidean distances in spikes/s between all possible pairs of the 104 tested human odorants were used to evaluate the spatial differences involved in the process of bed bug olfaction.

Of the 5356 pairs of human odorants tested, five of the top 10 closest pairs, which showed smallest Euclidean distance, were structurally and chemically fell into the same categories (Table 1). The top 10 odorant pairs that were farthest apart in the odorant space were found to all share one member: nonanal (Table 1). Although all the C5–C10 aldehydes (pentanal, hexanal, heptanal, octanal, nonanal and decanal) were very far away (≧100spikes/s) from almost all the other chemicals in different categories, especially the amine odorants (with a Euclidean distance ≧200spikes/s), nonanal was consistently farthest out. The three amine odorants were also a long way out (≧100spikes/s) from almost all the other chemical categories, especially the aldehyde odorants, with two exceptions: the heterocyclics thiazolidine and 1-methylpiperazine. These results suggest that both aldehydes (C_5_–C_10_) and amines are very important but mutually distinctive chemical components in human odorants for the chemoreception in bed bugs.

**Figure 6:**
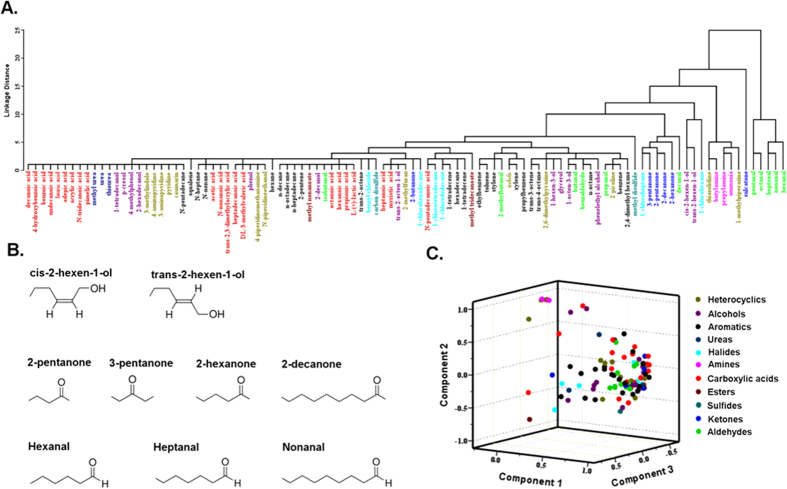
Primary presentations of odorant space among the olfactory sensilla. (**A**) Hierarchical cluster analysis for human odorants based on the Euclidean distances between them. Odorants are color coded by chemical class. (**B**) Typical odorants with close chemical structure are clustered together in the Hierarchical cluster analysis. (**C**) Relationships among human odorants of the indicated chemical classes at a dose of 1:10^2^ v/v revealed by PCA. Odorants are color coded by chemical class as in Fig. 6A. In PCA, vectors quantifying the responses of the 6 antennal sensilla to each tested odor are projected onto a three-dimensional region. Each axis represents the normalized neuronal responses of the olfactory sensilla in a new coordinate system determined by PCA. This three-dimensional representation captures 87.67% of the variation in the original 6-dimensional data set.

To visualize the relationships among odorants in this space, a hierarchical cluster analysis was performed on the odorants based on the responses of each olfactory sensillum. We found that odorants in the same chemical group often, though not always, clustered together (Fig. 6A). Particullarly, certain structurally similar molecules are observed to be tightly clustered, for example, cis-2-hexen-1-ol and trans-2-hexen-1-ol; 2-pentanone, 3-pentanone, 2-hexanone and 2-decanone; hexanal, heptanal and nonanal (Fig. 6B).

As another way of analyzing the relationships among odors, principle component analysis (PCA) was used to represent the six-dimensional odor space in a three-dimensional odor space. As in the hierarchical cluster analysis, odorants of aldehydes (green dots) or amines (pink dots) were more likely to cluster together but mutually highly separated (Fig. 6C). Acids were the most dispersive chemical groups in this odor space and some intermingling was observed in the odor space among odors of different classes (Fig. 6C). These results indicate that chemical class is one of the critical factors that are involved in determining the pattern of activation among olfactory sensilla on bed bug antennae.

### Identification of putative odorant receptors and co-receptor in the common bed bug

**Figure 7:**
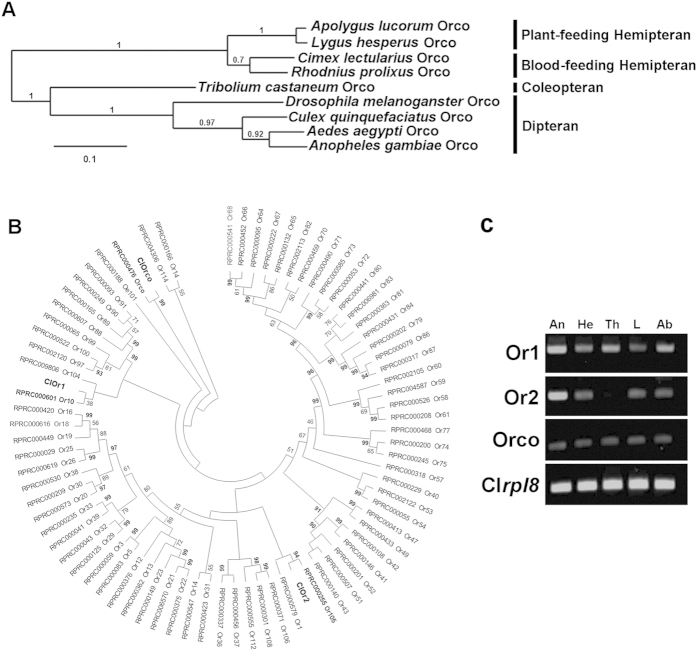
Phylogenetic analysis of ClOrco and ClOrs and their tissue-specific expression in the common bed bug. (**A**) Phylogenetic analysis of ClOrco with Orco orthologs from other insect species. The tree was constructed with MEGA6 based on a Clustal alignment of the amino acid sequences and selected Orco sequences from eight other insects. Numbers above individual branches indicate the percentage of 1,000 bootstrap replication trees in that branch. The scale bar indicates 10% divergence. The accession numbers for each Orco of insect species are: KC881255.1 for *Apolygus lucorum*; JQ639214.1 for *Lygus lineolaris*; RPRC000476; *Rhodnius proxilus*; CLEC006196 for *Cimex lectularius*; AM689918 for *Tribolium castaneum*; XM_001359327 for *Drosophila pseudoobscura*; XM_001651376 for *Aedes aegypti*; AY843205 for *Anopheles gambiae*; DQ231246.1 for *Culex quinquefaciatus*. (**B**) Phylogenetic analysis of ClOrs. The phylogenetic tree shows the relationships of two ClORs and ClOrco to their equivalents in *Rhodnius proxilus*. The tree was rooted with 76 odorant receptors of *R. proxilus* from the Vectorbase. Numbers above branches represent the percentage of 1,000 bootstrap replication trees in that branch, with only those above 50% shown. The ClOrco is clearly clustered with RpOrco, with 99% bootstrap support; ClOr2 is clustered with RpOr105, with 94% bootstrap support; and ClOr1 is clustered with RpOr10, although with only 38% bootstrap support. There is no support for the backbone of the relationships within the branches. (**C**) Tissue-specific expression profiles for ClOr1, ClOr2 and ClOrco. The house-keeping gene, Cl*rpl*8, was selected as the control in the semi-quantitative PCR of different tissue of bed bug. The symbols above the gel picture represent antennae (An), head (He), thorax (Th), legs (L) and abdomen (Ab), respectively.

The availability of the bed bug genome sequence allowed us to annotate two putative odorant receptors and their co-receptors and obtain the cDNA sequence. The blast results of the putative odorant receptors and co-receptors indicated different degree of sequence similarity with the odorant receptors in other insect species. Specifically, the odorant receptor co-receptor (Orco) is considered to be highly conserved, both in sequence and function, among different insect species^23^. In our study, the bed bug Orco shared 88%, 84%, and 83% of its identity in the amino acid sequences with another three Hemiptera species, *Rhodnius proxilus*, *Apolygus lucorum* and *Lygus Hesperus*. The phylogeny analysis for Orcos among different species also indicated that the Orco of the bed bug (ClOrco) is most likely to be clustered with the Orcos of *Rhodnius proxilus* (RpOrco), *Apolygus lucorum* (AlOrco) and *Lygus hesperus* (LhOrco) rather than other insect species such as mosquitos and fruit flies (Fig. 7A). Moreover, the ClOrco was found to be most likely to be clustered with RpOrco, possibly due to the common blood-feeding adaption in these two hemipterans while the other two hemipterans (*Apolygus lucorum*, *Lygus hesperus*) are plant-feeding.

We then assessed the relationships of these putative bed bug Ors (ClOrs) with 76 Ors from *R. proxilus* (RpOrs) by generating a phylogeny tree and conducting a bootstrap analysis (Fig. 7B). The results indicate that there is very strong evidence that ClOrco and RpOrco, specifically ClOr2 and RpOr105, are clustered together with a supportive value of over 90%, which suggests that they may share a common ancestor in the process of evolution. However, there is no strong evidence that ClOr1 is clustered with RpOrs, which suggests that ClOr1 may represent a bed bug-specific odorant receptor. The transcript levels of these three bed bug olfactory genes in different tissues were semi-quantitatively investigated with RT-PCR. Both Or1 and Or2 were much more highly expressed in the antennae compared with other olfaction-unrelated tissues, which suggests that both olfactory genes may play important roles in the chemoreception of bed bugs (Fig. 7C). The bed bug Orco is also more likely to be evenly expressed in the different tissue parts of the bed bugs, which also makes sense given that Ors that are involved in processes other than olfaction will also need Orco to function^24^.

### Functional characterization of putative olfactory receptors in the common bed bug

**Figure 8:**
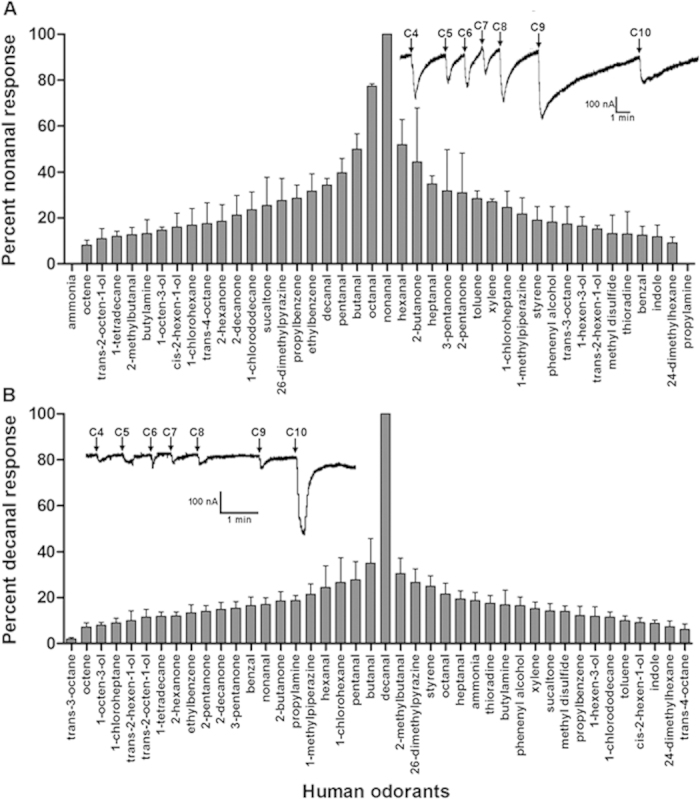
Current responses of ClOr1/Orco and ClOr2/Orco expressed in the *Xenopus* oocyte with two-electrode voltage clamp recording. (**A**) Oocytes expressing ClOr1/Orco were perfused with a panel of odorant compounds, eliciting a firing rate ≥50 spikes/s in single or multiple sensilla on the bed bug antennae. Each odorant was applied at a concentration of 10^−4^ M for 10 sec with immediate washes until the residue effect of the odorant was totally eradicated. All responses are normalized to the response of the same oocyte to 10^−4^ M nonanal (mean ± SEM, N = 4–5). The strongest responses are in the center and the weaker responses near the edges of the column graph. Representative current response traces elicited by C_4_–C_10_ aldehydes are on the right. (**B**) Oocytes expressing ClOr2/Orco were perfused with the same panel of odorant compounds as applied for ClOr1/Orco. All responses are normalized to the response of the same oocyte to 10^−4^ M decanal (mean ± SEM, N = 4–5). The strongest responses are again in the center and the weaker responses near the edges of the column graph. The representative current response traces elicited by C_4_–C_10_ aldehydes are on the left.

To further decipher the molecular mechanisms involved in the electrophysiological responses of the olfactory receptor neurons to human odorants, these two putative olfactory receptors (Or1, Or2) and their olfactory receptor co-receptor (Orco) were functionally tested using the *Xenopus* expressing system. The electrical responses of individual oocytes expressed with specific ClOr/ClOrco to different human odorants were recorded using a two-electrode voltage clamp. The human odorants that elicited firing rates ≥50 spikes/s on different olfactory sensilla of the bed bugs were chosen to perfuse the oocytes. The results showed that oocytes expressed with ClOr1/Orco displayed a wide range of current responses to human odorants at the concentration of 10^−4^ M tested in the perfusion (Fig. 8A). In particular, aldehydes stimulated the ion channel of ClOr1/Orco very strongly, with the strongest responses observed from nonanal and octanal, both of which were very efficient in eliciting neural responses in the single sensillum recordings (Fig. 8A). However, ClOr2/Orco showed a very narrow spectrum in response to the human odorants tested, even though aldehyde chemicals were still the most efficient ligands in stimulating this ion channel. Moreover, unlike ClOr1/Orco, which are likely generalists in detecting human odorants, ClOr2/Orco tend to be specialists and hence show much stronger responses to decanal than the other human odorants in the experiment (Fig. 8B).

**Figure 9:**
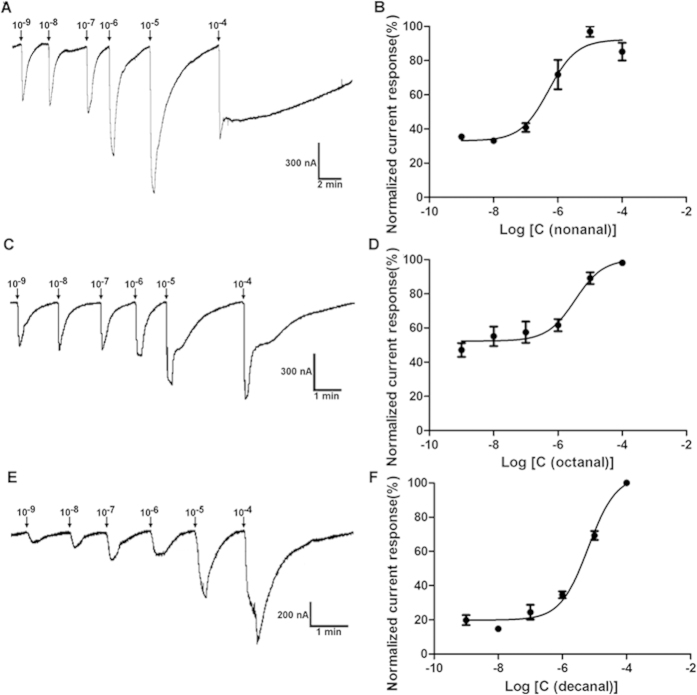
Dose-dependent responses of bed bug ClOr1/Orco and ClOr2/Orco to nonanal/octanal and decanal, respectively. (**A**) Current response traces of oocytes expressed ClOr1/Orco when challenged with nonanal at doses from 10^−9^ to 10^−4^ M; (**B**) Fitted dose-response curve from the current responses presented in Fig. 9A (Mean ± SEM, N = 4–6); (**C**) Current response traces of oocytes expressed ClOr1/Orco when challenged with octanal at doses from 10^−9^ to 10^−4^ M; (**D**) Fitted dose-response curve from the current responses presented in Fig. 9C (Mean ± SE/M, N = 4–6); (**E**) Current response traces of oocytes expressed ClOr2/Orco when challenged with decanal at doses from 10^−9^ to 10^−4^ M; (**F**) Fitted dose-response curve from the current responses presented in Fig. 9E (Mean ± SEM, N = 4–6). Each concentration of odorants was applied for 10 sec with immediate washes until the residue effect of the odorant was totally eradicated. Responses were normalized by defining the maximal response as 100.

When 10-fold sequential dilutions of nonanal/octanal and decanal (10^−9^ to 10^−4^ M) were applied in stimulating the ClOr1/Orco and ClOr2/Orco, respectively, the current response was clearly dose-dependent (Fig. 9A,C,E). The threshold for the ClOr1/Orco response to nonanal/octanal is also relatively lower than the ClOr2/Orco response to decanal (Fig. 9B,D,F). The EC50s of ClOr1/Orco for nonanal and octanal are 5.186 × 10^−7^ M and 3.398 × 10^−6^, respectively, and the EC50 of ClOr2/Orco for decanal is 6.424 × 10^−6^ M. We also found that in the dose response curves, relatively high initial current responses were elicited from low odorants concentration, which may result from the high binding affinity or efficiency of these chemical ligands to the ORs.

## Discussion

Bed bugs rely heavily on blood from their host, either human or animal, for survival and development and the neural responses of bed bug antennae to human odorants provide the primary messages that enable them to identify a potential blood source. Previous studies have tended to emphasize the importance of heat and carbon dioxide in attracting bed bugs, and studies that have focused on the role of human odorants in the process of host seeking have been very limited^11^. This study provides a systematic description of the neural responses of the olfactory antennal sensilla of bed bugs to 104 human odorants, and elucidates the different response profiles of the olfactory sensilla to various human odorants. Our results revealed that bed bugs exhibited neural responses to at least 42 human odorants with firing rates higher than 50 spikes/s, which suggests that at least at the olfactory sensillum level, bed bugs are sensitive to a number of human odorants, which included several aldehydes(C7–C10) and one ketone (sulcatone) that have been used in the behavior assay and showed more attracting to the bed bug at low concentration while more repelling at high concentration^11^.

Traps that combine CO_2_, heat and chemical lures have been tested in the lab, but the results revealed no significant additive effects of the chemical lure on the number of bed bugs captured compared to traps consisting of CO_2_ and heat alone^25^,^26^. The major components of these chemical lures are carboxylic acids, which are known attractants for blood-feeding insects such as mosquitoes, biting midges, kissing bugs and tsetse flies^27^. However, in our study we found that bed bugs showed no neural responses to any of the carboxylic acids tested. Therefore, our finding may partially explain why no additive effects were observed in the bed bug catches when carboxylic acids were added to the traps.

Our results also revealed that the amine chemicals exhibited the most significant difference with the aldehydes in the chemoreception process. This huge differentiation in the chemoreception may result from the distinctive expression of two different types of olfactory receptors in the olfactory neurons of D type sensilla and C type sensilla, respectively. In this study, we characterized the function of two Ors in response to several aldehydes (octanal, nonanal and decanal), but not amines. These results were fairly consistent with our findings from the neuron recording, which showed that nonanal was the strongest stimulant for neurons housed in the Dα, Dβ and Dγ sensilla of bed bugs, with firing rates of 248 ± 34, 213 ± 25, and 223 ± 25 spikes/s, respectively. Octanal is also very effective in eliciting neural responses in the Dα, Dβ and Dγ sensilla, with firing frequencies of 135 ± 23, 200 ± 12, and 162 ± 25 spikes/s, respectively, while decanal also elicited good responses on the Dα, Dβ and Dγ sensilla, with firing rates of 74 ± 9, 85 ± 6, and 108 ± 22 spikes/s, respectively. It therefore seems likely that these two Ors are expressed in neurons housed in the Dα, Dβ and Dγ sensilla. However, we also found that the amines tested in our study were exclusively recognized by the neurons housed in the grooved-peg C type olfactory sensilla of bed bugs. This result is consistent with the findings reported in previous studies on mosquitoes (*Culex quinquefasciatus*) and kissing bug (*Triatoma infestans*), where the grooved-peg sensilla also showed very strong responses to the amine chemicals^28^,^29^. Some recent studies uncovered a new family of insect chemo-receptors, ionotropic receptors (IRs), which were proved to be responsible for the recognition to polar molecules, like the amines^30^,^31^,^32^. Interestingly, IRs have been widely reported in the coeloconic sensillum in *Drosophila melanogaster*^30^,^31^. For the bed bugs, as grooved peg C sensillum shared similar cuticle and pore structure with the coeloconic sensillum of other insects^16^ and amines are exclusively detected by the C sensilla, we proposed that probably IRs may express in the neurons housed in C type olfactory sensillum, which are playing a key role in responding to amine chemical in the environment.

Previous studies have indicated that insect ORNs fall into two classes, specialists and generalists, according to their electrophysiological studies^33^,^34^,^35^, with specialists responding to only a very limited number of odors with particular biological relevance, like pheromones, while generalists respond to a much wider range of odors. In the malaria vector, *Anopheles gambiae,* a number of Ors (Or5, Or31, Or1, Or26) have been identified that respond to only a single odorant, while other Ors respond to a chemically very diverse set of odorants^36^. These narrowly tuned Ors have been found to respond with high affinity to compounds related to *An. gambiae* biology, including host seeking cues from human sweat. Our study has revealed that in the common bed bug, Or1/Orco was more likely to be a generalist as it showed a more general response to a wide variety of odorants, while Or2/Orco tended to be a specialist with a particularly strong response to decanal alone. Indeed, decanal is not only a major component of human emanations, it has also proved to be an important compound in the bed bug aggregation pheromone^37^. It is important to note, however, that this dichotomy theory has been challenged by recent advances in the functional analysis of Ors, especially systematic studies of large number of Ors in mosquitoes and fruit flies^20^,^36^ and it has been suggested that insect Ors actually present a continuum of tuning breadths in response to odorants, rather than a neat dichotomy. We therefore expect that as more and more bed bug Ors are functionally elucidated, a similar continuum model of tuning breadths will emerge.

## Materials and Methods

### Insects, scanning electron microscopy, and single sensillum recording

The *C. lectularius* colony was a gift from Dr. Haynes (University of Kentucky, Lexington, KY). For the single sensillum recordings, adult bed bugs were used throughout. The bed bugs were reared at 25 ± 2 °C under a photoperiod of 12:12 (L: D). Scanning Electron microscopy (SEM) and single sensillum recording (SSR) experiments were conducted as described by Liu *et al.* (2014). Briefly, the bed bugs (male or female) were anaesthetized (2–3 min on ice) and mounted on a microscope slide (76 × 26 mm) between 2 pieces of double-sided tape. The antennae were fixed by double-sided tape to a cover slip resting on a small ball of dental wax to facilitate manipulation. The cover slip was placed at an appropriate angle to the bed bug head. Once mounted, the bed bug was placed under a LEICA Z6 APO microscope and the antennae examined at high magnification (×720). Two tungsten microelectrodes were each sharpened in 10% KNO_2_ at 2–10 V to a ~1 μm tip diameter; the reference electrode, connected to ground, was inserted into the abdomen of the bed bug and the other electrode, connected to a preamplifier (10×, Syntech, Kirchzarten, Netherlands), was inserted into the shaft of olfactory sensillum to complete the electrical circuit in order to extracellularly record the olfactory receptor neuron (ORN) potentials^38^. Controlled manipulation of the electrodes was performed using 2 micromanipulators (Leica, Germany). The preamplifier was connected to digital signal converter (IDAC, Syntech, Netherlands) and thence to the computer for signal recording and visualization. Signals were recorded for 10 s starting 1 s before stimulation at a sampling rate of 96000/s, and the action potentials were counted off-line for 500 ms before and after stimulation. Changes in spike rates during the 500 ms pre-stimulation period were subtracted from the activity recorded during the 500 ms stimulation period and the difference converted to the conventional scale of spikes/s.

### Stimulation and stimuli

Based on Bernier’s (2000) GC-MS study on emanations from human skin^39^, 104 commercially available human odorants from 11 chemical groups (carboxylic acids, esters, aldehydes, alcohols, ketones, aliphatics/aromatics, halides, heterocyclics, amines, sulfides and ureas) were used in the study (Table S2). Each of the human odorants was diluted in dimethyl sulfoxide (DMSO) to a stock solution with a concentration of 1:10 v/v (100 μg/μl). Subsequently, serial decadic dilutions (i.e., successive 1/10 dilutions) were made from the stock solution for each of the chemicals. Ten microliters of each dilution were dispersed on a filter paper (2 × 10 mm) that was then inserted in a Pasteur pipette to create each stimulus cartridge. A pipette containing solvent alone served as the control. A constant airflow across the antenna was maintained at 20 ml/s throughout the experiment. Purified and humidified air was delivered to the preparation through a glass tube (10 mm inner diameter). The glass tube was perforated by a small hole, slightly larger than the tip of the Pasteur pipette, 10 cm away from the end of the tube. Stimulation was achieved by inserting the tip of the stimulus cartridge into the hole of the glass tube. A stimulus controller (Syntech, Germany) diverted a portion of the air stream (0.5 1/min) to flow through the stimulus cartridge for 500 ms, thus delivering the stimulus to the sensilla. The distance between the end of the glass tube and the antennae was ≤1 cm. All the human odorants were tested on each type of antennal sensillum at least 6 times each and the value of spikes/s obtained by averaging all the recordings for each sensillum to each odorant. Those sensilla that failed to show a response of firing rate of 15 spike/s, were considered to be non-responders^40^.

### Tissue-specific semi-quantitative RT-PCR

One hundred adult bed bugs were randomly collected for harvesting different tissue parts from the whole body, including antennae (An), head (He), thorax (Th), legs (L) and abdomen (Ab). All tissues were stored at −80 °C until use. Total RNAs from different tissues were extracted using the acidic guanidine thiocyanate-phenolchloroform method^41^. Five μg of total RNA was treated with a DNA-free Kit (Ambion) and cDNA synthesised from 0.5 μg DNase-treated RNA using Superscript III Reverse Transcriptase System (Invitrogen) in a total volume of 20 μl. PCR amplification for bed bug odorant receptor genes ClOr1 and ClOr2 and odorant receptor co-receptor gene ClOrco were performed, respectively, using primer pairs listed in Table S3. The house-keeping gene Clrpl8 is used as the control in semi-quantitative PCR of different tissues of bed bugs with the amplification primer pair listed in Table S3 42.

### Expression of putative odorant receptor and co-receptor genes in the *Xenopus* oocyte system and two-electrode, voltage-clamp electrophysiological recordings

The entire coding regions of the putative olfactory receptor genes ClOr1 and ClOr2 and co-receptor gene ClOrco were amplified using the primers listed in Table S4 with a cutting site and Kozak sequence added. The purified PCR products were digested with *Not*I-HF/*Nhe*I-HF (New England Biolabs, MA) and then cloned into pT7Ts vector (a gift from Dr. Wang in Institute of Plant Protection, CAAS), with a Kozak sequence added behind the cutting site in the forward primer. The constructed vectors were linearized with *EcoR*I-HF and cRNAs synthesized from the linearized vectors with mMESSAGE mMACHINE T7 (Ambion, Carlsbad, CA). Mature healthy oocytes (stage V–VII) (Nasco, Salida, CA) were treated with collagenase I (GIBCO, Carlsbad, CA) in washing buffer (96 mM NaCl, 2 mM KCl, 5 mM MgCl_2_, and 5 mM HEPES [pH = 7.6]) for about 1 h at room temperature. After being cultured overnight at 18 °C, oocytes were microinjected with either 5 ng cRNAs of both Ors and Orco or sterilized double-distilled water. The water-injected oocytes were set as the control. After injection, oocytes were incubated for 4–7 days at 18 °C in 1× Ringer’s solution (96 mM NaCl, 2 mM KCl, 5 mM MgCl_2_, 0.8 mM CaCl_2_, and 5 mM HEPES [pH = 7.6]) supplemented with 5% dialyzed horse serum, 50 mg/ml tetracycline, 100 mg/ml streptomycin and 550 mg/ml sodium pyruvate. Whole-cell currents were recorded from the injected *Xenopus* oocytes with a two-electrode voltage clamp. Human odorants induced currents were recorded with an OC-725C oocyte clamp (Warner Instruments, Hamden, CT) at a holding potential of −80 mV. Human odorants were selected and dissolved in dimethyl sulfoxide (DMSO) to 1 M Stock solutions and stored at −20 °C. Before testing, each stock solution was diluted with 1× Ringer’s buffer. Data acquisition and analysis were carried out with Digidata 1440A and pCLAMP 10.2 software (Axon Instruments Inc., CA). Dose-response data were analyzed by GraphPad Prism 5.0 (GraphPad Software Inc, CA).

### Data analysis

Hierarchical cluster analysis and principle component analysis (PCA) for the odorant space were performed using PASW 18.0 (IBM, NY). Euclidean distance and between-group linkage classification methods were used for the hierarchical cluster analysis^20^,^43^. PCA was conducted using the correlation matrix. A value of *P* ≤ 0.05 was considered statistically significant.

## Additional Information

**How to cite this article**: Liu, F. and Liu, N. Human Odorant Reception in the Common Bed Bug, *Cimex lectularius*. *Sci. Rep.* **5**, 15558; doi: 10.1038/srep15558 (2015).

## Supplementary Material

Supplementary Information

## Figures and Tables

**Table 1:** Euclidean Distance (ED) of the top ten closest and farthest odorant pairs in the olfaction space of bed bugs.

Closest odorant pairs	ED(Spikes/s)	Farthest odorant pairs	ED(Spikes/s)
Decanoic acid/4-hydroxybenzoic acid	3.6	Nonanal/Propylamine	406.2
Urea/Tridecanoic acid	4.1	Nonanal/Methylpyrazine	405.3
Methylindole/Benzoic acid	4.4	Nonanal/Butylamine	394.9
Decanoic acid/Methylindole	4.5	Nonanal/Ammonia	390.6
Urea/Acrylic acid	4.6	Nonanal/Thiozilidine	386.6
Pentadecane/Decanoic acid	4.6	Nonanal/ethylurea	380.7
Decane/Octadecane	4.7	Nonanal/Perperidinemethamine	380.3
Undecanoic acid/4-hydroxybenzoic acid	5.0	Nonanal/Phenol	380.3
Pimelic acid/Tridecanoic acid	5.1	Nonanal/Methylvaleric acid	380.2
Adipic acid/Tridecanoic acid	5.1	Nonanal/Lauric acid	378.5
