# Supplementary material for: Human Odorant Reception in the Common Bed Bug, Cimex lectularius
Source: Sci Rep. 2015 Nov 2;5:15558. doi: 10.1038/srep15558 (PMC4629130; doi:10.1038/srep15558)
Supplement: Supplementary Information [file srep15558-s1.pdf]

Prepared for Publication in

Scientific Report

Address Correspondence to:

Dr. Nannan Liu

Department of Entomology & Plant Pathology

301 Funchess Hall, Auburn University

Auburn, AL 36849-5413, USA

Phone: [001] (334) 844-2661;

Fax: [001] (334) 844-5005

E-mail: liunann@auburn.edu

Human Odorant Reception in the Common Bed Bug, *Cimex lectularius*

Abbreviated title: Human Odorant Reception in the Common Bed Bug

Feng Liu, Nannan Liu*

Department of Entomology and Plant Pathology, Auburn University, Auburn, AL 36849, USA

*Corresponding author

Acknowledgements: The project described was supported by Award AAES Hatch/Multistate Grants ALA08-045 and ALA015-1-10026 to N.L. The authors declare no competing financial interests.

Table S1. Firing rates of neuronal responses of different olfactory sensilla in bed bug antennae to human odorants (n=6~10, Mean ± SEM)

Odorants	Dα±SEM	Dβ±SEM	Dγ±SEM	C±SEM	E1±SEM	E2±SEM
**acetic acid**	16.67±2.31	35.33±7.17	15.00±2.30	17.33±2.01	1.50±1.37	4.33±0.98
**propionic acid**	20.67±1.67	36.00±1.39	23.33±4.49	-1.33±2.57	-0.50±0.77	13.20±1.98
**hexanoic acid**	19.75±5.58	30.86±4.18	29.67±6.62	7.00±2.46	1.60±0.51	4.40±1.07
**heptanoic acid**	10.57±1.89	48.29±2.71	14.33±3.63	15.33±2.13	4.00±0.80	4.33±1.47
**octanoic acid**	10.80±2.62	31.20±4.10	24.50±2.55	4.40±1.54	5.33±0.62	15.60±2.07
**N-nonanoic acid**	9.14±1.44	22.00±3.91	22.67±3.73	19.20±5.70	3.33±0.80	5.00±1.00
**decanoic acid**	9.14±1.76	17.00±1.40	16.00±4.27	11.67±1.20	3.50±1.35	7.25±1.50
**lauric acid**	8.00±2.06	15.50±2.75	8.67±2.67	6.40±1.79	2.57±0.78	4.22±0.83
**N-tridecanoic acid**	10.29±2.19	21.75±1.33	15.20±2.82	7.60±0.83	3.56±0.91	6.25±1.13
**myristic acid**	14.22±2.79	48.00±4.96	9.56±2.11	16.40±2.79	2.00±0.53	8.67±3.46
**N-pentadecanoic acid**	8.86±1.99	18.29±2.16	23.09±3.16	12.40±2.43	2.00±0.40	49.00±4.40
**heptadecanoic acid**	6.00±0.80	10.00±1.39	21.33±4.19	18.00±1.60	1.20±0.44	8.00±1.60
**acrylic acid**	15.00±1.60	22.44±1.52	11.20±1.38	7.20±1.98	2.57±0.19	8.80±1.12
**undecanoic acid**	14.57±2.38	16.86±3.04	13.20±2.40	12.80±2.50	3.33±1.07	7.00±1.00
**benzoic acid**	9.14±1.67	15.50±1.45	18.91±4.32	8.33±1.24	2.50±0.80	4.75±1.38
**adepic acid**	12.67±2.49	22.75±3.98	12.67±2.79	10.80±2.18	5.00±0.93	5.25±1.43
**pimelic acid**	6.33±1.47	22.29±1.93	14.22±2.25	5.60±0.51	3.33±0.53	8.57±1.24
**4-hydroxybenzoic acid**	11.75±1.15	15.00±2.40	16.89±3.08	12.80±4.54	3.00±1.07	7.40±1.52
**L-(+)-lactic acid**	25.56±3.36	32.25±2.38	24.29±2.87	6.40±2.49	2.29±0.39	2.33±2.23
**DL-3-methylvaleric acid**	5.33±1.67	14.00±2.26	9.43±2.30	24.67±1.22	3.50±0.77	3.60±1.15
**trans-2,3-dimethylacrylic acid**	7.33±0.92	32.00±1.13	15.25±2.96	20.00±1.39	-1.50±1.65	7.20±1.54
**propanal**	7.50±0.40	40.67±3.33	36.67±2.80	29.33±4.82	7.50±1.51	2.33±0.44
**butanal**	35.50±3.02	51.00±7.32	43.33±3.30	24.67±6.99	4.00±0.57	8.57±1.63
**pentanal**	140.86±12.87	183.00±8.67	167.11±8.24	14.00±2.82	2.75±1.10	6.86±1.45
**hexanal**	220.22±13.63	180.00±14.20	186.25±5.50	22.00±4.38	6.67±1.42	6.67±1.65
**heptenal**	218.50±8.04	214.33±13.09	208.80±14.51	33.71±5.07	12.40±0.88	5.67±1.20
**octanal**	135.00±9.18	200.00±4.62	161.71±9.95	17.33±2.01	29.50±4.45	10.00±1.33
**nonanal**	248.50±13.95	212.67±10.18	223.43±9.97	21.00±3.60	16.40±2.29	4.67±1.60
**decanal**	74.50±3.6	85.33±2.44	107.67±8.94	8.67±1.85	13.20±3.38	5.00±1.33
**Isobutanal**	14.00±1.73	30.00±4.00	32.29±4.63	13.33±3.23	4.00±1.13	9.67±1.73
**2-methylbutanal**	38.20±4.38	69.00±6.4	63.00±4.80	13.00±1.03	2.86±1.08	8.50±2.45
**benzaldehyde**	19.80±3.9	55.25±2.83	41.11±5.83	22.00±4.20	5.33±0.95	4.25±1.45
**p-cresol**	6.86±1.53	19.71±2.61	10.25±2.33	7.67±1.24	3.60±1.41	6.67±1.01
**4-methylphenol**	13.67±2.00	17.71±1.67	12.75±2.10	1.67±0.67	3.00±0.67	9.25±1.90
**1-hexen-3-ol**	38.80±3.36	71.50±4.20	46.44±4.56	8.00±2.58	5.14±0.85	11.78±2.31
**cis-2-hexen-1-ol**	153.56±11.00	62.22±2.92	28.57±4.15	5.60±1.04	2.29±0.85	9.14±1.59
**trans-2-hexen-1-ol**	113.33±16.10	49.78±3.40	21.25±4.01	10.00±2.95	7.20±1.54	5.00±2.00
**trans-2-octen-1-ol**	8.86±2.95	52.00±7.12	20.29±2.97	16.80±4.82	4.80±0.90	8.80±1.40

**2-decanol**	21.50±3.19	40.50±3.44	31.00±5.18	6.67±2.07	3.20±0.70	5.60±1.00
**phenelethyl alcohol**	26.00±3.55	51.25±4.46	44.00±2.92	9.60±3.01	7.60±1.73	6.40±1.00
**glycerol**	13.33±2.36	73.00±4.46	41.33±9.50	4.40±1.64	11.00±3.80	4.80±1.10
**phenol**	9.00±1.39	8.57±2.64	10.67±2.13	21.43±4.18	-1.50±1.77	5.00±1.87
**1-tetradecanol**	7.75±1.13	12.50±2.35	13.40±2.59	7.33±1.16	6.00±1.07	14.40±2.62
**2-hexadecanol**	10.25±1.70	17.80±2.32	21.50±4.40	8.40±2.07	3.50±1.00	14.33±4.68
**1-octen-3-ol**	36.00±4.93	59.67±5.48	45.00±3.80	31.43±6.29	8.00±1.60	7.33±0.62
**hexane**	24.50±3.22	14.00±2.26	30.67±4.40	15.50±2.86	2.50±0.40	11.00±1.47
**N-heptane**	14.00±2.21	33.43±5.42	21.25±5.17	15.33±4.22	3.60±1.09	7.20±0.38
**n-octane**	22.89±2.27	53.25±2.30	31.25±3.70	15.60±1.54	4.33±1.20	7.67±1.38
**N-nonane**	12.86±4.33	25.14±3.66	20.00±4.42	22.67±5.39	3.50±0.60	5.50±1.24
**n-decane**	14.20±2.64	33.50±4.05	21.14±2.81	4.00±1.52	2.40±0.51	8.86±2.30
**2,4-dimethyl hexane**	15.60±2.75	34.25±3.68	56.00±6.40	15.33±2.61	2.33±1.02	4.33±1.92
**N-pentadecane**	7.00±1.40	17.71±1.73	15.50±3.00	15.60±3.71	2.67±0.89	7.80±1.20
**hexadecane**	8.33±2.58	30.00±2.67	14.67±2.37	5.20±1.54	-3.60±0.58	34.89±3.50
**n-heptadecane**	16.22±2.94	35.00±3.50	25.00±4.50	5.67±1.55	5.33±0.89	12.33±3.18
**n-octadecane**	13.33±2.25	29.71±3.98	19.50±5.20	6.00±1.13	2.29±0.42	8.00±2.99
**benzene**	15.33±4.89	42.00±3.39	32.33±7.13	24.67±3.61	3.00±1.03	5.00±1.07
**ethylbenzene**	22.67±2.51	55.00±6.31	68.86±5.64	16.00±3.27	-6.00±1.20	8.00±1.80
**propylbenzene**	25.67±2.34	40.25±5.60	84.00±1.90	18.00±5.94	7.60±2.43	7.50±2.60
**stylene**	21.00±2.12	72.00±5.25	81.43±7.98	18.00±5.60	6.80±1.84	6.33±1.73
**squalene**	15.00±1.66	14.50±3.49	19.14±2.73	8.33±2.84	-8.80±1.02	6.33±1.40
**toluene**	21.67±2.55	62.57±7.24	65.14±6.21	12.00±2.95	5.20±1.02	6.00±2.49
**xylene**	18.00±4.45	50.67±1.22	64.86±7.23	23.33±1.22	13.50±4.50	6.29±1.96
**2-pentene**	15.00±3.28	32.00±4.20	29.71±3.33	2.00±0.80	3.60±0.83	6.67±1.20
**trans-2-octene**	9.33±1.8	41.33±5.80	35.20±5.72	5.00±0.84	2.67±0.89	5.71±0.64
**trans-3-octene**	35.33±2.96	71.60±6.40	34.67±4.33	4.57±1.51	3.71±0.39	6.57±1.00
**trans-4-octene**	30.75±2.30	69.78±6.12	29.56±3.04	14.00±3.43	5.00±1.47	5.50±2.08
**1-hexadecene**	10.22±1.70	43.78±3.50	12.57±2.78	5.67±1.38	2.00±0.46	41.67±5.29
**1-tetradecene**	14.73±2.16	38.33±5.51	13.67±2.53	13.00±2.62	4.29±1.31	60.00±5.63
**methyl tridecanoate**	22.67±5.79	24.67±2.31	28.67±5.08	18.00±0.80	22.50±4.54	31.33±2.93
**methyl nonanoate**	19.67±3.01	34.25±6.53	29.50±3.14	8.00±2.09	6.00±2.24	10.00±2.13
**2-butanone**	12.29±3.21	59.71±7.80	10.75±3.28	23.33±5.58	6.00±1.60	3.00±1.00
**2-pentanone**	36.00±4.45	102.00±4.80	59.00±5.40	29.33±4.89	7.00±3.55	4.00±1.33
**2-hexanone**	50.00±4.07	138.00±9.12	110.75±6.10	7.14±1.95	9.25±1.18	3.50±1.26
**2-decanone**	30.73±4.73	100.00±5.20	86.75±4.78	9.60±2.29	9.71±1.01	11.14±2.39
**3-pentanone**	46.18±5.17	122.67±4.80	59.00±4.64	6.67±1.87	5.67±1.20	14.67±2.41
**sulcatone**	24.67±5.76	40.00±7.16	226.33±7.36	12.86±2.65	5.67±1.20	3.33±1.10
**1-chloroheptane**	61.33±4.50	131.43±3.53	76.22±2.94	12.33±4.39	8.67±1.60	4.25±0.79
**1-chlorododecane**	18.80±2.78	33.71±4.24	21.43±4.38	5.20±0.72	6.33±0.76	57.33±9.49
**lauryl chloride**	10.57±1.51	17.75±3.64	10.50±2.21	-9.20±2.09	14.00±2.00	58.80±6.00
**1-chlorotetradecane**	12.33±1.63	25.75±3.81	17.71±2.71	17.33±2.61	1.60±0.51	39.20±4.60
**1-chlorohexadecane**	17.40±2.80	39.00±3.07	21.75±2.75	23.00±4.10	3.14±0.62	20.33±1.38

**1-chlorohexane**	136.00±13.59	146.25±12.59	41.33±5.74	23.20±5.44	-7.20±2.05	14.50±0.40
**benzyl chloride**	8.86±2.57	21.50±5.22	37.14±4.88	6.80±2.01	2.80±0.83	13.60±1.90
**propylamine**	3.50±1.20	28.00±3.32	22.00±3.20	194.57±15.93	4.00±1.73	5.33±0.98
**butylamine**	5.00±1.03	14.67±1.73	18.00±2.49	144.00±12.06	6.00±1.96	-0.33±0.67
**ammonia**	8.50±0.40	34.00±3.39	48.00±6.14	200.00±6.97	1.00±2.96	2.57±0.78
**carbon disulfide**	6.57±1.51	28.67±4.70	9.56±1.31	-7.67±1.63	5.60±1.47	9.60±1.60
**methyl disulfide**	6.00±2.09	94.00±18.35	7.67±1.38	5.67±1.38	4.50±1.40	7.67±1.40
**methyl urea**	6.29±1.04	10.00±1.33	11.56±2.61	10.80±1.22	3.14±0.98	5.20±1.38
**thiourea**	6.00±0.69	10.25±1.53	16.22±3.65	5.20±0.90	2.50±0.80	10.57±0.95
**urea**	13.14±1.67	23.75±2.90	14.50±2.35	6.00±1.28	2.33±0.44	6.89±1.64
**N-piperidineethanol**	7.71±2.14	18.57±3.05	14.50±3.44	30.67±5.78	3.50±1.40	5.50±1.60
**1-methylpiperazine**	16.29±4.65	10.00±2.73	12.25±3.51	176.80±16.34	-4.50±1.00	2.80±0.53
**2-methylfuran**	11.67±0.79	47.56±5.13	19.71±2.33	9.33±2.38	3.71±1.04	8.40±1.24
**thiazolidine**	5.33±0.79	21.71±2.33	18.57±3.51	129.60±14.30	5.60±1.41	4.00±1.07
**3-methylindole**	6.00±1.60	14.86±2.35	17.33±2.96	9.60±2.37	3.33±0.89	6.89±2.00
**3-aminopyridine**	11.14±2.12	21.78±2.23	11.33±2.31	4.80±1.34	4.00±0.53	11.25±2.58
**4-aminopyridine**	10.33±1.73	21.75±1.30	10.75±1.58	11.20±2.62	4.00±0.27	9.40±1.52
**pyridine**	7.14±1.31	25.14±2.22	23.25±1.78	8.40±1.15	2.25±0.73	8.40±1.60
**2,6-dimethylpyrazine**	18.00±1.60	74.00±6.72	56.00±5.69	10.40±1.41	4.86±0.72	7.40±2.27
**coumarin**	5.14±1.89	19.14±1.53	20.00±2.60	1.00±0.40	3.75±1.30	9.20±1.28
**4-piperidinemethanamine**	4.67±0.46	5.00±0.57	18.86±3.54	21.33±7.85	3.00±2.12	7.60±2.30
**2-picoline**	6.00±1.79	39.75±5.17	41.43±4.10	18.00±5.08	7.67±1.73	4.00±0.71
**indole**	12.00±1.60	51.67±5.93	70.57±5.52	28.57±3.54	5.33±1.20	6.29±0.85
**DMSO**	1.64±0.48	2.89±0.49	2.44±0.55	2.86±0.43	1.20±0.38	1.00±0.44

Table S2 Human odorants lists used in this study

Chemicals*	CAS	Company	Purity	Behavior Activity	SSR Activity	Reference
**Carboxylic acids**						
acetic acid	64-19-7	Sigma	99%			
propionic acid	79-09-4	Fisher	99.5	+		[1, 2]
hexanoic acid	142-62-1	Sigma	99.5		+	[3]
heptanoic acid	111-14-8	Sigma	96			
octanoic acid	124-07-2	Sigma	98			
N-nonanoic acid	112-05-0	Sigma	97%			
decanoic acid	334-48-5	Sigma	98			
lauric acid	143-07-7	Sigma	99			
N-tridecanoic acid	638-53-9	Sigma	98			
myristic acid	544-63-8	Sigma	98			
N-pentadecanoic acid	1002-84-2	Sigma	99			
heptadecanoic acid	506-12-7	Sigma	98			
acrylic acid	79-10-7	Acros Organics	99			
undecanoic acid	112-37-8	Sigma	98			
benzoic acid	65-85-0	Sigma	99.5			
adepic acid	124-04-9	Acros Organics	99			
pimelic acid	111-16-0	Acros Organics	98			
4-hydroxybenzoic acid	99-96-7	Acros Organics	99			
L-(+)-lactic acid	79-33-4	Sigma	98	+	+	[1, 2, 3]
DL-3-methylvaleric acid	105-43-1	Acros Organics	97			
trans-2,3-dimethylacrylic acid	80-59-1	Acros Organics	98			
**Aldehydes**						
propanal	123-38-6	Sigma	97			
butanal	123-72-8	Sigma	99			
pentanal	110-62-3	Sigma	97			
hexanal	66-25-1	Sigma	98			
heptenal	111-71-7	Sigma	92	+	+	[4]
octanal	124-13-0	Sigma	99	+	+	[4]
nonanal	124-19-6	Aldrich	95	+	+	[4, 5]
decanal	112-31-2	Sigma	98	+	+	[4, 5]
isobutanal	78-84-2	Sigma	99			
2-methylbutanal	96-17-3	Sigma	90			
benzaldehyde	100-52-7	Sigma	99	+	+	[3, 5]
**Alcohols**						
p-cresol	106-44-5	Acros Organics	99			
4-methylphenol	123-07-9	Acros Organics	97		+	[3]
1-hexen-3-ol	4798-44-1	Sigma	98			
cis-2-hexen-1-ol	928-94-9	Aldrich	95			

trans-2-hexen-1-ol	928-95-0	Acros Organics	96			
trans-2-octen-1-ol	18409-17-1	Acros Organics	98			
2-decanol	1120-06-5	Sigma	98			
phenelethyl alcohol	60-12-8	Sigma	99			
glycerol	56-81-5	Sigma	99			
phenol	108-95-2	Sigma	99		+	[3]
1-tetradecanol	112-72-1	Sigma	97			
2-hexadecanol	14852-31-4	Sigma	99			
1-octen-3-ol	3391-86-4	Aldrich	99	+	+	[1, 2, 3]
**Aromatics and Aliphatics**						
hexane						
N-heptane	142-82-5	Sigma	99			
n-octane	111-65-9	Sigma	98			
N-nonane	111-84-2	Fisher	100			
n-decane	124-18-5	Fisher	99			
2,4-dimethyl hexane	589-43-5	Fidher	99			
N-pentadecane	629-62-9	Acros Organics	99			
hexadecane	544-76-3	Acros Organics	99			
n-heptadecane	629-78-7	Sigma	99			
n-octadecane	593-45-3	Sigma	99			
benzene	71-43-2	Sigma	99.8			
ethylbenzene	100-41-4	Sigma	99			
propylbenzene	103-65-1	Sigma	98			
styrene	100-42-5	Sigma	99			
squalene	111-02-4	Sigma	98			
toluene	108-88-3	Sigma	99.8			
xylene	106-42-3	Sigma	99.5			
2-pentene	109-68-2	Aldrich	99			
trans-2-octene	13389-42-9	Aldrich	97			
trans-3-octene	14919-01-8	Aldrich	98			
trans-4-octane	14850-23-8	Aldrich	98			
1-hexadecene	629-73-2	Aldrich	99			
1-tetradecene	1120-36-1	Aldrich	97			
**Esters**						
methyl tridecanoate	1731-88-0	Acros Organics	97			
methyl nonanoate	1731-84-6	Acros Organics	95			
**Ketones**						
2-butanone	78-93-3	Sigma	99.7		+	[3]
2-pentanone	107-87-9	Fisher	99			
2-hexanone	591-78-6	Fluka	96			
2-decanone	693-54-9	Aldrich	98			
3-pentanone	96-22-0	Fisher	99			
sulcatone	110-93-0	Sigma	98	+	+	[3, 4, 5]

**Halides**					
1-chloroheptane	629-06-1	Aldrich	99		
lauryl chloride	112-52-7	Acros Organics	99		
1-chlorotetradecane	2425-54-9	Acros Organics	98		
1-chlorohexadecane	4860-03-1	Aldrich	95		
1-chlorohexane	544-10-5	Fisher	95		
benzyl chloride	100-44-7	Sigma	99		
**Amines**					
propylamine	107-10-8	Aldrich	99		
butylamine	109-73-9	Aldrich	99.5		
ammonia	7664-41-7	Aldrich	100	+	[3]
**Sulfides**					
carbon disulfide	75-15-0	Fisher	99.9		
methyl disulfide	624-92-0	Sigma	99		
**Ureas**					
methyl urea	598-50-5	Sigma	97		
thiourea	62-56-6	Sigma	99		
urea	57-13-6	Sigma	99		
**Heterocyclics**					
N-piperidineethanol	3040-44-6	Acros Organics	99		
1-methylpiperazine	109-01-3	Acros Organics	99.5		
2-methylfuran	534-22-5	Acros Organics	99		
thiazolidine	504-78-9	Acros Organics	98		
3-methylindole	83-34-1	Acros Organics	98		
3-aminopyridine	462-08-8	Acros Organics	99		
4-aminopyridine	504-24-5	Acros Organics	98		
pyridine	110-86-1	Acros Organics	100		
2,6-dimethylpyrazine	108-50-9	Acros Organics	96		
coumarin	91-64-5	Acros Organics	99		
4-piperidinemethanamine	7144-05-0	Acros Organics	97		
2-picoline	109-06-8	Acros Organics	98		
indole	120-72-9	Aldrich	99	+	[3]
DMSO	67-68-5	Sigma	100		

*Human odorants were selected referring to the study of Bernier et al., 2000. All the human odorants were tested at the dose of 1:100 v/v, with the exception of dimethyl sulfoxide (DMSO), which was used as the solvent and 100% of DMSO was used as the control. Numbers refer to published behavioral studies or SSR studies of specific human odorant on the bed bugs: [1] Anderson et al. (2009); [2] Wang et al. (2009); [3] Harraca et al. (2010); [4] Harraca et al. (2012); [5] Siljander et al. (2008).

Table S3 Primers used in the semi-quantitative tissue-specific PCR of bed bug odorant receptors and co-receptor

Genes	Forward primer	Reverse primer
ClOr1	TCATGTGCCAGGTCACTTATAC	CGAGCAGAGAATCGGAACAA
ClOr2	CCTTTCCCGTGGAGTTCATTA	CTGAATAGAGCAAGAGCGTAGAG
ClOrco	TTACAGCCTCTGATGGAGTTAAG	AGTGGGATGTTGGATGAAGTAG
Cl*rpl*8*	AAAGGCACGGTTACATCAAAGGTG	TAGTCTTGAACCTATAGGGGTCCC

*Cl*rpl*8 is the house-keeping gene used as the control in semi-quantitative PCR of different tissues of bed bugs (Zhu et al., 2012).

Table S4 Primers used in cloning the full length of bed bug odorant receptors and co-receptor

Genes	Forward primer*	Reverse primer*
ClOr1	ccggctagcgccaccATGATGTGGAAAGTAGCGAG	ctaggcggccgcCAATTTAGAAGACACCGTCT
ClOr2	ccggctagcgccaccATGGGAACTGTAAAAACAGA	ctaggcggccgcACCCATGAGGGCTTTGAGTA
ClOrco	ccggctagcgccaccATGCAGAAAGTAAAGATGCA	ctaggcggccgcCTAACTCTTGAGCTGCACCA

*pink color: protective nucleotides; red color: restriction enzyme cutting site; green color: Kozak sequence; black color: gene specific primer sequence
